# Nursing evaluation of pediatric preoperative anxiety: a qualitative study

**DOI:** 10.1590/1518-8345.6230.3738

**Published:** 2023-03-06

**Authors:** Carmen Jerez Molina, Laura Lahuerta Valls, Victoria Fernandez Villegas, Susana Santos Ruiz

**Affiliations:** 1 Campus Docent Sant Joan de Déu, School of Nursing, Barcelona, Spain; 2 Hospital Sant Joan de Déu, Nursing Department, Ambulatory Surgery, Barcelona, Spain; 3 Hospital Sant Joan de Déu, Nurse Research Department, Barcelona, Spain

**Keywords:** Preoperative Care, Perioperative Nursing, Preoperative Assessment, Anxiety, Child, Qualitative Research, Cuidados Pré-Operatórios, Enfermagem Perioperatória, Avaliação Pré-Operatória, Ansiedade, Criança, Pesquisa Qualitativa, Cuidados Preoperatorios, Enfermería Perioperatoria, Evaluación Preoperatoria, Ansiedad, Niño, Investigación Cualitativa

## Abstract

**Objective:**

to explore and describe how perioperative nurses assess and interpret the child’s behavior before entering the operating room, identifying the strategies they use to reduce anxiety and the proposals for improvements.

**Method:**

descriptive qualitative study using semi-structured interviews and participant observation of daily routines. Thematic analysis of data. This study follows the recommended criteria for publication of articles of the qualitative methodology Consolidated Criteria for Reporting Qualitative Research.

**Results:**

four topics emerged from the data: a) assessment of anxiety or close communication with the child and their family; b) evaluating what was observed; c) managing anxiety and d) improving the assessment or proposals for improvements in daily practice.

**Conclusion:**

nurses assess anxiety in their daily practice through observation using their clinical judgment. The nurse’s experience is decisive for the appropriate assessment of the preoperative anxiety in child. Insufficient time between waiting and entering the operating room, lack of information from child and their parents about the surgical procedure, and parental anxiety make it difficult to assess and properly manage anxiety.

## Resumo

**Objetivo:**

explorar e descrever como as enfermeiras perioperatórias avaliam e interpretam o comportamento da criança antes de entrar na sala de cirurgia, identificando as estratégias que utilizam para minimizar a ansiedade e as propostas de melhoria.

**Método:**

estudo qualitativo descritivo utilizando entrevistas semiestruturadas e observação participante das rotinas diárias. Análise temática dos dados. O estudo segue os critérios recomendados para publicação de artigos da metodologia qualitativa *Consolidated Criteria for Reporting Qualitative Research*.

**Resultados:**

quatro temas emergiram dos dados: a) avaliação da ansiedade ou comunicação próxima com a criança e sua família; b) analisando o que foi observado; c) controlando a ansiedade e d) melhorando a avaliação ou propostas de melhoria na prática diária.

**Conclusão:**

as enfermeiras avaliam a ansiedade em sua prática diária por meio da observação e usando julgamento clínico. A experiência da enfermeira é decisiva na avaliação adequada da ansiedade pré-operatória da criança. A falta de tempo entre a espera e o momento de entrar na sala de cirurgia, a escassez de informação que a criança e os pais têm sobre o processo cirúrgico e a ansiedade dos pais, dificultam a avaliação e o controle adequado da ansiedade.

## Resumen

**Objetivo:**

explorar y describir cómo las enfermeras perioperatorias evalúan e interpretan el comportamiento del niño antes de entrar a quirófano, identificando las estrategias que utilizan para minimizar la ansiedad y las propuestas de mejora.

**Método:**

estudio cualitativo descriptivo mediante entrevistas semiestructuradas y observación participante de las rutinas diarias. Análisis temático de los datos. El estudio sigue las recomendaciones de criterios para la publicación de artículos de metodología cualitativa *Consolidated Criteria for Reporting Qualitative Research*.

**Resultados:**

cuatro temas surgieron de los datos: a) evaluación de la ansiedad o comunicación estrecha con el niño y su familia; b) valorando lo observado; c) manejando la ansiedad y d) mejorando la evaluación o propuestas de mejora para la práctica diaria.

**Conclusión:**

enfermeras evalúan la ansiedad en su práctica diaria de forma observacional utilizando el juicio clínico. La experiencia de la enfermera es determinante en la adecuada evaluación de la ansiedad prequirúrgica del niño. La falta de tiempo entre la espera y el momento de entrar a quirófano, la mala información que tiene el niño y los padres sobre el proceso quirúrgico y la ansiedad de los padres dificultan la evaluación y el manejo correcto de la ansiedad.

**Highlights**

(1) Assessment of pediatric preoperative anxiety.

(2) Management of pediatric preoperative anxiety.

(3) Appropriate information on the procedure for both parents and children.

(4) Low reliability of the current assessment of pediatric preoperative anxiety.

(5) Necessary involvement of surgical administrators.

## Introduction

Anxiety is a psychological reaction that can be observed in health users who are about to undergo a surgery. A surgical intervention can be very stressful, especially for children and their families, which can cause high levels of preoperative anxiety and postoperative behavioral changes^1^. Recent studies report a prevalence of anxiety of 67-75% in children aged 2-12 years^2^ ^-^ ^3^. Age, personality, developmental status and previous experiences can be triggering factors^2^, and high parental anxiety can also influence the child’s anxiety^4^.

It is known that an effective reduction in preoperative anxiety can improve the child’s cooperation with the care team^3^, promote a better postoperative response^5^, increase parental satisfaction with the procedure and improve the quality of care^6^. In children, strategies such as the administration of anxiolytic medication, the use of videos, hospital clowns, music therapy and allowing the presence of their parents/caregivers during anesthetic induction are some of the interventions that aim to reduce anxiety and, thus, promote a more cooperative child during anesthetic induction^7^ ^-^ ^9^. However, we cannot only focus on reducing the child’s anxiety, as their parents can also be the cause of their children’s anxiety, as we have already mentioned. Therefore, strategies such as music, clowns, preopertative programs and educational materials have also been shown to reduce parental anxiety^10^, thereby contributing to a holistic and comprehensive care, which is centered on the children and their family.

Child- and family-centered care can help to humanize the surgical process, which must be centered not only on the child, but also on the family and on the relationship established between them and the health professional. In this approach, the family is an active part of the surgical process and the need for information is increasingly important, parents need information about the process and children must be involved to answer their questions, consider their fears, turn their attention to others and talk to them^11^ ^-^ ^12^. A recent study demonstrates how a child- and family-centered program decreases the administration of preoperative sedation, increases the satisfaction of parents and health professionals, and decreases the anxiety of parents and children, in addition to not modifying the surgery times^13^.

On the one hand, it is known that the causes of preoperative anxiety are multiple and its effects can last up to months after the surgical intervention^14^, and on the other hand, it is known that there is a high prevalence of children with preoperative anxiety^15^. Both statements should lead us to think about how we are assessing anxiety and how we are going to minimize anxiety and act accordingly. The assessment of preoperative anxiety in the daily routine is currently performed through the clinical judgment by health professionals^16^. However, we have not found in the literature how health professionals interpret the behaviors and actions of the child before entering the operating room. The objective of this study was to explore and describe how perioperative nurses assess and interpret the child’s behavior before entering the operating room, identifying the strategies they use to minimize anxiety and proposals for improvements.

## Method

### Design

A descriptive qualitative study. The qualitative design was considered as the most appropriate to know how the nurses in their daily practice assess the child’s preoperative anxiety as it provided the researchers with a rich descriptive content from the perspective of the study subjects^17^ ^-^ ^18^. The manuscript has been prepared according to COREQ checklist (Consolidated Criteria For Reporting Qualitative Research)^19^.

### Place of study

The study was carried out in the pediatric outpatient surgery unit of a hospital in Barcelona. A third-level University Hospital specialized in the health of children and pregnant women and the first pediatric center in Spain to implement an Outpatient Surgery Unit. The unit has 23 armchairs/individual beds with enough space for two accompanying persons (father/mother) to be with the child during the preparation and after the surgical procedure. Four surgical procedures are performed (two in the morning and two in the afternoon), which can be in the specialty of surgery, ophthalmology, traumatology, otorhinolaryngology, dermatology and dentistry, depending on the surgical schedule.

### Study period

Interviews and participant observation were carried from October 2018 to January 2019. 

### Population 

It was proposed the participation of all perioperative nurses from the outpatient surgery unit of the two shifts, about 15 nurses. All nurses in charge of care for children who were admitted to the unit on the day of their surgical intervention. Participants were selected by maximum variation sampling^20^, which ended when data saturation was reached^21^. Two evaluators decided by consensus when the data saturation was reached. The participants were chosen taking into account that they worked in the unit and were in charge of care for child and, therefore, for the assessment of anxiety before the surgical intervention; and that there was representation of nurses from both shifts (morning and afternoon). The final sample included nine participants. The first author invited personally and individually all the nurses of the unit to participate. One of the selected nurses who met the eligibility criteria did not agree to be interviewed and/or observed in their daily practice for personal reasons. 

### Data collection

Semi-structured interviews and participant observation were conducted. The interviews, which lasted about thirty minutes, were conducted in a distraction-free office in the surgical unit by the main author, who worked as a research nurse for a year. The researcher had knowledge in conducting interviews because of previous studies and her academic career. To start the interview, nurses were asked to visualize the moment they first contacted the child and their parents in the waiting room. Once the moment was visualized, a boy or a girl in particular, the first question was introduced: What do you see in the child that makes you think that they have preoperative anxiety? From this first question, the following ones were asked according to the main objective. Thus, questions related to anxiety and how each nurse assessed anxiety, used non-pharmacological strategies to reduce it, and proposed future strategies for its management and assessment were asked (Figure 1).

**Figure 1:** Guide for semi-structured interviews

When do you assess child’s anxiety?, How do you assess child’s anxiety that makes you think they are nervous? (Child Anxiety Assessment Process)
What techniques do you use to reduce preoperative anxiety? On what occasions have you had to reduce parental anxiety? (Difficulties and barriers to reduce anxiety in clinical practice)
What do you think should be done to improve the child’s anxiety management and how do you think it could be done? (Improvements to reduce children’s anxiety)

During the interviews, permission was requested for audio recording and field notes were taken, which were used at the end of the interviews to summarize the conversation, clarify some answers and provide additional information. As an additional technique and to increase the quality of the data, participant observation was also performed. It was considered necessary to observe the nurses during their assessment of anxiety, as it is a complex phenomenon to be assessed only through personal interviews^22^. A structured observation was proposed, as it is only possible when the researcher has sufficient information and knowledge about the phenomenon under study, and it was carried out through a event sampling and involved the selection of the events to be observed^23^. Thus, the first contact with the child and their parents and the assessment and management of anxiety by the nurse were observed. The observations were carried out during ten non-consecutive days in both shifts and in different times of the day. 

### Data analysis

The proposed thematic analysis^24^, was carried out in line with the recommendations and consisted of six phases: a) familiarization: the interviews and field notes collected during the participant observation were transcribed. Next, the data were read and reread and the information was redefined taking into account semantic cohesion, b) codes creation: the most relevant data characteristics were coded and each code was conceptually defined, c) search for themes: the codes were grouped and four themes emerged, d) revision of themes: the two evaluators reviewed the themes to reach an agreement and prepare a thematic map, e) definition of themes: the agreed definitions of each theme were carried out and finally, f) elaboration of the report: selection of examples of excerpts from the interview and the observations. Final analysis of the selected excerpts was carried out according to their relation with the research question and the literature. 

The observations recorded in the field diary, the interview transcripts and the notes obtained throughout the study were analyzed by peers.

### Criteria of rigor and ethical considerations

The Ethics and Clinical Research Committee of the hospital approved the study (código 15-2018). All participants were informed about their voluntary participation and signed a consent form. 

The recommended reliability criteria were considered to achieve methodological rigor^25^. The participants received the transcripts and, later, the results. There were no changes in this regard. The verbatim reports of the interviews were used to illustrate the results to ensure reliability. The observation of daily practice and the field notes were analyzed for data triangulation. To ensure a greater objectivity in data analysis, it was carried out by peers, with the intervention of the author who did not know the participants and the surgical environment, together with the main author, an expert perioperative nurse. A complete description of the context where the data were collected was carried out to enable their transfer to other contexts.

Total anonymity of responses and personal data was achieved in accordance with the current legislation on the protection of personal data.

## Results

The average age of the participating nurses was 36.6 years (Standard deviation 10.9). All of them were women with an average length of service of 9.8 years in the unit. Sample characteristics are shown in Table 1.

**Table 1:** Demographic characteristics of participants. Barcelona, Spain, 2019

Participant	Age (years)	Length of service (years)
1	40	11
2	27	1
3	25	1
4	48	21
5	50	21
6	38	4
7	28	4
8	24	1
9	50	25

Note: All participants were women. Length of service refers to the years the nurse had been working in the Outpatient Surgery Unit and, therefore, assessing the child’s preoperative anxiety before entering the operating room.

To illustrate the results, verbatim reports of the interviews or excerpts of the field notes have been chosen. The names of participants have been replaced by the letter “P” (participant), followed by a number and the letter “O”, which means a field diary entry during the observation. The interviews lasted an average of 30 minutes.

**Figure 2:**
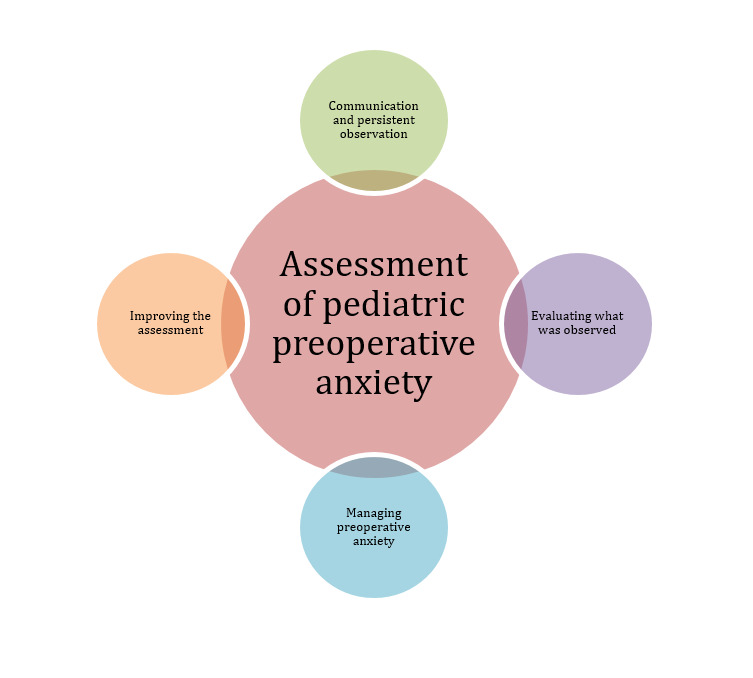
Child- and family-centered care during the assessment of pediatric preoperative anxiety

Four main themes emerged from the analyzed data: assessment, evaluation, management of anxiety and proposals for improving anxiety assessment (Figure 2).

### Theme 1: Communication and persistent observation of the child and their family or assessment of pediatric preoperative anxiety

Communication and persistent observation of the child and their family were identified as key components in the assessment of the child’s anxiety before entering the operating room. The nurses begin the assessment of the children’s anxiety when they first contact them and their parents: While the nurse places the child and his parents in the environment, she observes their behaviors and actions. This persistent observation continues over time until the nurse is sure of her assessment: *I walk out and wait two seconds to call the child and I look from the outside how they are in the waiting room, so if I notice that they are agitated or something like that, and they are very restless... I already get worried, of course, because if they are already like this in the waiting room, in the operating room the situation will be …. even more turbulent. If they are very quiet, that also catches my attention, so when I take them down the hallway... I also observe how they behave... And once inside the room, depending on the questions I ask them and everything else... because sometimes I have to ask, because some of them don’t know what they’re coming for, no, they don’t know they’re coming for surgery, so I notice them even more nervous.* (P: 3)

### Theme 2: Evaluating what was observed

While they observe the behaviors, actions and verbal and non-verbal communication of the child and their parents with each other or with the health team, the nurse can get an idea of the anxiety, fears and concerns regarding the surgical intervention of the children and their accompanying persons.

Some of the observed behaviors warn the nurses that the child is very nervous: *They grab on to their parents, they cry, they hide, and based on how they respond when you ask them, you can notice if they are receptive, if they enjoy when you offer them to paint.* (P:2)

In older, shy or very emotional children, it is very difficult to assess anxiety, as they can hide their nervousness, either because they do not show it or because they are collaborative and participative: *It’s the nervousness that makes them express themselves like that, very euphoric…they jump, play, they breathe inside the mask and they participate…so as soon as they come in, they panic. There are also those who are very withdrawn… it is necessary to be careful with those too… when it’s their first time or not, if they know where they are going, and obviously, there are many factors.* (P:5)*.* In these cases, or when there is any doubt as to whether the child is expressing preoperative anxiety through their behavior, the nurse asks the parents if they notice that their child is nervous. Sometimes, the parents’ assessment of their child’s anxiety is wrong, as they notice their child as nervous as on other occasions. The nurse then doubts about her own criteria, which delays the nursing assessment of anxiety. Excerpt from a observation note: *The nurse asked the parents if they noticed that their child was nervous. The parents responded that the kid is like that <the child is like that>*. (O of a four-year-old boy)

The children’s lack of information about the surgical process and the short time to prepare the child are detected on several occasions as the cause of high anxiety during anesthesia induction: *All that joy and euphoria turns into a terrifying fear (...) so as soon as they come in (for anesthesia induction), they panic.* (P:5)

*The children didn’t receive sufficient information about what they were going to do with them, that is, they were unprepared from home, in addition, we don’t have too much time either, however, I can even give them information, but the children also need information given by their parents.* (P:6)

### Theme 3: Managing pediatric preoperative anxiety

This category is about those strategies that nurses put into action through communication and observation of the child and their parents to reduce anxiety in both. When the nurse detects that the child has anxiety, she immediately acts to minimize it: providing information about the procedure appropriate to the child’s age and cognitive level, offering distracting activities (stories, drawings to color, games, etc.) or relying on other professionals (clowns and volunteers). Considering that when entering the operating room, the child will find out that they have to breathe inside the mask, the nurse, in addition to providing information, insists that they practice with her how to do it in order to become familiar with it. It was observed that sometimes the face mask was impregnated with strawberry essence to avoid the child to reject it: *The nurse has asked for a face mask from the operating room (the one that the girl will wear later). She explains how to put it on her face after the girl’s refusal to put the mask on herself. The nurse teaches the process to her using the doll that the girl had in her hands. When the mask was close to the doll, the girl realized that the mask had a known odor and not the odor that she supposed. The eight-year-old girl relaxed and began to practice how to breathe inside the mask while the nurse was telling her that when she is with her mom in the operating room, the doctor would place a tube, and that she would have to breathe into it to fall asleep while her mom would be with her*. (O of a six-year-old girl)

In addition, nurses use words appropriate for the child’s cognitive age, avoiding others that can cause more stress, anxiety or fear. Nurses involve parents in this process, instructing and explaining about the anesthetic induction process to them. *(….) information, that they will have to breathe inside a mask like the one on airplanes, that they will fall asleep, that we will cure them and when they wake up they will be cured. And they will soon go back to their mom and dad when they are awake again, they will go to a room with a nurse and then they will go back to mom and dad. As soon as I see them, I go look for a mask (…) and I explain to them that they can come in with their mom or dad (….). I never say operate, I say heal. It depends on the child, when I realize that they will get already scared if I tell them that they will fall asleep, I tell them in another way…* (P:4)

During the observation sessions, it was noticed that some parents also lack information about the surgical process. The nurse must, in such cases, provide parents with information. In such cases, the nurse must take a moment to be alone with the parent who will accompany their child during the anesthetic induction: *The nurse asked the child if they wanted to go to the restroom. The child went to the restroom accompanied by their father, and the nurse seized the moment with the mother to explain to her alone (without the child’s presence), in more detail, the moment of entering the operating room: what will happen, where the child will be sitting, how they should act in the operating room and what behaviors she will observe in the child when they are unconscious*. (O of a 6-year-old boy) 

Sometimes the strategies used by the nurses to reduce anxiety do not have the expected result, so it is necessary to inform the anesthesiologist. This outcome is sometimes frustrating for the nurse, since the efforts she made and the actions she used to reduce the child’s anxiety were ineffective and the last choice to get a cooperative child during anesthetic induction is to give preoperative sedation. This alternative solution to manage preoperative anxiety, when non-pharmacological alternatives have failed, causes concern among nurses, since the anesthesiologist does not arrive in time so that the sedation has the desired effect on the child before entering the operating room, and it is not desirable to receive sedation to avoid side effects of the drug: *Yes, you’ve done everything, but something tells you that when they go into the operating room, they will be even more nervous…. So, I wait for the anesthesiologist and I tell him that the child needs premedication, that they are very nervous, with anxiety.* (P:5)*. There are some anesthesiologists who do not want the children to receive preoperative sedation because they say there is no enough time, or whatever the reason. New anesthesiologists are more receptive.* (P:1)

The observation confirmed that the first child in the surgical program is the most difficult to assess, estimate and develop an effective management of anxiety, as the surgical intervention begins when the anesthesiologist arrives, so if the child is very nervous and the strategies are non-pharmacological, they do not have the wanted effect. In addition, the problem is heightened by the fact that the first children in the session are usually the youngest ones and least receptive to non-pharmacological interventions: *A nurse says that the work pace in the afternoon shift is faster, the children are scheduled with a short space of time between them and sometimes, we do not have enough time to explain the process or teach them how to put the mask.* (P:3)

### Theme 4: Improving the assessment of pediatric preoperative anxiety or future proposals for the management of pediatric preoperative anxiety

This category includes those proposals that nurses have observed in their daily practice, which could improve the assessment and management of pediatric preoperative anxiety. The nurses proposed that parents should also help with their child’s surgery preparation, and this would also help them to lessen their own anxiety, and consequently, lessen that of their children: *Before the child comes in the operating room, you can give as much information as possible to the parents, to the children, so that they are in agreement at home, right? It´s important that the parents explain to them what the process will be like, what they will have to do.* (P:8)*.* In addition, they suggest that parents should be instructed during the process not only providing them with information, but also so that with this information they can help their children face the day of surgical intervention: *Sometimes children have anxiety because their parents have anxiety. There are children who come without knowing why they come and that also influences them. In addition, sometimes the parents have not told them the truth because they are the ones who are nervous.* (P:2)

When the child is very young, or when it is not possible to understand them due to their age or pathology, nurses claim the need for a protocol that unifies the decisions and that preoperative sedation medication is prescribed in advance in consultations: *A boy with behavioral problems was admitted during the observation period, his mother stated that the boy had already undergone surgery on other occasions and that he became very nervous and that on some occasion he had been given medication to calm him down (O a 4-year-old boy).* The interviewed nurses suggested that the preoperative sedation guidance should be standardized from the anesthesiologist’s consultation: *Evaluate anxiety, we already do that, it’s fine, but as for the medication, we should have something standardized from anesthesia, or something like that, or a paper that gives us authorization to pre-medicate (…) to perform everything more or less in the same way…* (P:5)

## Discussion

The objective of this study was to know how perioperative nurses interpret the child’s behavior and how they act accordingly in their daily practice. 

In this study, the assessment of child’s anxiety before entering the operating room was carried out by the nurses in charge of preoperative care by observing behaviors and actions. Other studies have also reported that the operating room staff are the ones in charge to assess and interpret the child’s behavior through clinical judgment^26^ ^-^ ^28^. However, such assessment is prone to an important subjective error, as it depends on the skill of the observer to interpret the behaviors and the time available to observe^16^. 

Our study suggests that nurses interpret the child’s behaviors to assess anxiety and that, if there are doubts about their interpretation, parents are asked. However, the assessment of preoperative anxiety in children by trained personnel has been shown to be more accurate than that reported by parents^28^, although it is not sufficient to detect all children with preoperative anxiety^16^. 

In care services with high levels of pressure, such as those in the surgical field, there is a limited time to prepare patients. However, the time necessary to provide children and their parents with important preoperative information about postoperative pain, anesthesia, meal initiation, and discharge requirements should already be considered within this short preoperative time^29^. Parents need information and health literacy has been shown to reduce anxiety so that they can feel as an active part in the surgical process of their children^30^.

All the actions that the nurse takes to reduce preoperative anxiety, such as providing age-appropriate information and avoiding words that may cause more stress, anxiety or fear, aim at achieving a more cooperative child during anesthesia induction. The implementation of non-pharmacological strategies, such as information about the procedure, playing with the face mask and other distractions, is effective in reducing the child’s preoperative anxiety, however, sometimes these strategies are insufficient to help the child cope with the situation^31^. In these cases, it is necessary to notify the anesthesiologist to administer sedation to the child. However, as in other studies^32^, this work shows that decisions regarding the administration of preoperative sedation are based on personal criteria that lead to non-unified practices.

The results show that there is no protocol or preparation program for parents and children who will undergo outpatient surgery in the hospital studied. These preparation programs, suggested by the nurses studied, have already been pointed out in the literature as one of the aspects that would reduce the levels of anxiety in parents and children on the day of the surgical intervention^33^ and would save time in the surgical process^34^.

Training the use of the face mask at home and teaching distraction techniques to parents are two components of the preoperative preparation program observed as the most effective in keeping the child’s anxiety stable throughout all phases of the preoperative period on the day of the surgical intervention^35^. In addition, the child who is aware of the process is a facilitator and it is beneficial for the health professional, as the child can actively participate if they are informed^36^. Likewise, when the parents are informed about what is going to happen and about what is expected of them as parents, they feel involved in the care of their child and not as bystanders, as for example, during anesthetic induction^37^. Preoperative preparation of children and their parents could reduce the levels of anxiety, improve coping, and promote postoperative recovery^38^. The nurses in this study have stated that children and parents should be prepared before going to the operating room^39^. 

Although we currently have non-pharmacological strategies to reduce pediatric preoperative anxiety, we still have limitations^14^. As the surgical time to prepare children and parents is very short on the day of the surgical intervention, other strategies not related to therapeutic play could be proposed, since these have already proven to be effective^40^. Thus, these strategies would be supplemented with other ones, such as information on the process by team trained in emotional care^41^ and reinforced by means of written information^42^.

One of the limitations of this study is that the assessment was performed only in a pediatric outpatient surgery unit of a specific hospital. However, the triangulation of techniques such as systematic observation and interviews with both novice and expert nurses means that, although the results cannot be generalized, they can be considered in similar contexts to improve the assessment of pediatric surgical anxiety in daily practice.

This study contributes to the knowledge on the importance of perioperative nurses in the management of pediatric preoperative anxiety. Nursing care for child makes all the strategies used aim at a single objective: to minimize the child’s preoperative anxiety, as high levels of pre-operative anxiety in a child can have post-operative consequences that go beyond their stay in the hospital. Supervisors in these units should consider the need to focus preoperative nursing care on the child and their family, either by training parents to be facilitators in the surgical process of their children, or by providing the child with information appropriate to their age and stage of development. 

## Conclusion

The main results reveal that the determination of pediatric preoperative anxiety is based on clinical judgment by observing the child’s behaviors and actions. Lack of time time, parents’ level of anxiety and lack of information about the surgical process are the main difficulties faced by nurses in their daily practice. There are several strategies used to reduce anxiety, including distraction, clarification on the surgical process and age-appropriate communication.

Lack of time is the main barrier for strategies such as active listening, therapeutic play and age-appropriate information to reduce children’s anxiety before surgical intervention. It would be up to the facilitator to guide and train parents before their children’s surgical intervention so that they can be an active part of the process, as well as provide children with age-appropriate information through the implementation of preoperative preparation programs and parent training throughout the surgical process so that they can be an active part in this process. 

Although there is still a need to assess preoperative anxiety through the use of validated instruments that aim to unify the care and strategies based on the objective assessment of preoperative anxiety, nurses consider that perioperative care should focus on the child and their family, and therefore, the assessment of anxiety should be performed considering the child-parents and child-nurse relationships.

Based on the results found, future studies are recommended to assess whether the preoperative information provided by a team trained in pediatric emotional care reduces the child’s anxiety on the day of the surgical intervention, as well as to study whether the variation and difficulty in the assessment of preoperative anxiety in daily practice is homogenized by using preoperative anxiety assessment protocols that were unified by all health professionals involved in the perioperative process.
